# Relative toxicity of mood stabilisers and antipsychotics: case fatality and fatal toxicity associated with self-poisoning

**DOI:** 10.1186/s12888-018-1993-3

**Published:** 2018-12-27

**Authors:** Anne E. Ferrey, Galit Geulayov, Deborah Casey, Claudia Wells, Alice Fuller, Clare Bankhead, Jennifer Ness, Caroline Clements, David Gunnell, Navneet Kapur, Keith Hawton

**Affiliations:** 1 Centre for Suicide Research, Department of Psychiatry, University of Oxford, Warneford Hospital, Oxford, UK; 2 Office for National Statistics, Newport, UK; 3 Nuffield Department of Primary Care Health Sciences, University of Oxford, Oxford, UK; 4 Centre for Self-harm and Suicide Prevention Research, Derbyshire Healthcare NHS Foundation Trust, Derby, UK; 5 Centre for Suicide Prevention, University of Manchester, Manchester, UK; 6 School of Social and Community Medicine, University of Bristol, Bristol, UK

**Keywords:** Drug toxicity, Mood stabilisers, Antipsychotics, Suicide, Self-poisoning

## Abstract

**Background:**

Bipolar and other psychiatric disorders are associated with considerably increased risk of suicidal behaviour, which may include self-poisoning with medication used to treat the disorder. Therefore, choice of medication for treatment should include consideration of toxicity, especially for patients at risk. The aim of this study was to estimate the relative toxicity of specific drugs within two drug categories, antipsychotics and mood stabilizers, using large-scale databases to provide evidence that could assist clinicians in making decisions about prescribing, especially for patients at risk of suicidal behaviour.

**Method:**

Two indices were used to assess relative toxicity of mood stabilisers and antipsychotics: case fatality (the ratio between rates of fatal and non-fatal self-poisoning) and fatal toxicity (the ratio between rates of fatal self-poisoning and prescription). Mood stabilisers assessed included lithium [reference], sodium valproate, carbamazepine, and lamotrigine, while antipsychotics included chlorpromazine [reference], clozapine, olanzapine, quetiapine and risperidone. Fatal self-poisoning (suicide) data were provided by the Office for National Statistics (ONS), non-fatal self-poisoning data by the Multicentre Study of Self-harm in England, and information on prescriptions by the Clinical Practice Research Datalink. The primary analysis focussed on deaths due to a single drug. Cases where the drug of interest was listed as the likely primary toxic agent in multiple drug overdoses were also analysed. The study period was 2005–2012.

**Results:**

There appeared to be little difference in toxicity between the mood stabilisers, except that based on case fatality where multiple drug poisonings were considered, carbamazepine was over twice as likely to result in death relative to lithium (OR 2.37 95% CI 1.16–4.85). Of the antipsychotics, clozapine was approximately18 times more likely to result in death when taken in overdose than chlorpromazine (single drug case fatality: OR 18.53 95% CI 8.69–39.52). Otherwise, only risperidone differed from chlorpromazine, being less toxic (OR 0.06 95% CI 0.01–0.47).

**Conclusions:**

There was little difference in toxicity of the individual mood stabilisers. Clozapine was far more toxic than the other antipsychotics. The findings are relevant to prescribing policy, especially for patients at particular risk of suicidal behaviour.

## Background

Mood stabilisers and antipsychotics are used to treat psychiatric disorders such as bipolar disorder and schizophrenia. It is estimated that 5–8% of people with bipolar disorder die by suicide [1]. However, other studies suggest a higher rate [2, 3]. The equivalent figure for schizophrenia is 5% [4]. The method of suicide used by people with these disorders is often self-poisoning [5]. One important consideration when prescribing drugs for these conditions is their relative toxicity in overdose, because if patients self-poison they are likely to use their prescribed medication [6, 7]. Several mood stabilisers are also anticonvulsants used to manage epilepsy. Epilepsy is also associated with an increased risk of suicidal behaviour, including self-poisoning [8]. It is therefore important for clinicians be as well-informed as possible about the relative toxicity of drugs they prescribe for a given condition [9]. Previous work on the use of psychotropic medication in non-fatal self-poisoning in patients in Ireland suggested that more than 10% of the sample ingested antipsychotics, while approximately 7% used mood stabilisers [7]. Most of these drugs had been prescribed to the patient prior to the overdose. Establishing the relative toxicity of mood stabilisers and antipsychotics is important for clinical practice and regulatory agencies. The main aim of this study was to estimate the relative toxicity of specific drugs within two drug categories, antipsychotics and mood stabilisers, using large-scale databases. This will provide evidence that could assist clinicians in making decisions about prescribing, especially for patients at risk of suicidal behaviour.

## Methods

We investigated the relative toxicity of the mood stabilisers and antipsychotics most often used for fatal and/or non-fatal self-poisoning in the UK. We considered antipsychotics and mood stabilisers together because both are used as treatments for severe mental illnesses. We used two approaches: first, case fatality, in which the number of self-poisoning deaths involving a specific drug is compared to the number of episodes of non-fatal self-poisoning involving this drug [10, 11]; second, fatal toxicity, in which the number of deaths by self-poisoning involving a specific drug is compared to the number of individuals prescribed this drug [12]. We previously used these approaches to investigate the relative toxicity of antidepressants, [11] to highlight the toxicity of co-proxamol, [13] and to examine the relatively toxicity of benzodiazepines [14].

### Study drugs

The mood stabilisers investigated in this study were lithium and the anti-convulsants sodium valproate, carbamazepine and lamotrigine. The antipsychotics investigated included chlorpromazine, clozapine, olanzapine, quetiapine and risperidone. The period covered by the study was 2005–2012.

### Deaths

We obtained information on drug poisoning deaths receiving a verdict of intentional self-poisoning (ICD-10 codes X60–64) or death of undetermined intent (open verdict; ICD-10 codes Y10-Y14), that involved either mood stabilisers or antipsychotics, from the Office of National Statistics based on deaths in individuals aged 15 years and over occurring during 2005–2012 in England registered to the end of 2014. We included open verdicts as this is policy with regard to suicide statistics and research in the UK because a high proportion are thought to be probable suicides [15–17]. Intentional self-poisonings and open verdicts are henceforth termed ‘suicides’. We conducted two analyses. The first included only deaths involving single drugs (in the presence or absence of alcohol), while the second included these deaths together with deaths where the drug under investigation was the first drug recorded by the coroner in multiple drug overdoses, again with or without alcohol. This assumes that the first drug recorded was likely to have been the primary agent involved in the death. Death data were obtained for males and females separately for all drugs.

### Non-fatal self-poisoning

Data on non-fatal self-poisoning was obtained from the Multicentre Study of Self-harm in England [18]. The Multicentre Study of Self-harm collects data on all presentations for self-harm to the emergency departments at five general hospitals. These include one hospital in Oxford, three hospitals in Manchester and two hospitals in Derby that merged into one in 2009. Self-harm is defined as intentional self-poisoning or self-injury, irrespective of the motivation or degree of suicidal intent, [19] and self-poisoning includes the intentional ingestion of more than the prescribed amount of any drug. This applies whether or not there is evidence that the act was intended to result in death. Data were collected on gender, age, date and method of self-poisoning, including the specific drugs ingested. Episodes of non-fatal self-poisoning include all non-fatal overdoses involving the mood stabilisers and antipsychotics under investigation, regardless of whether or not other drugs or alcohol were involved.

We extracted data for all episodes of non-fatal self-poisoning involving individuals aged 15 years and over which included the mood stabiliser or antipsychotic under investigation. The analysis included only episodes of self-harm by individuals who resided in the catchment area of Oxford City, The City of Manchester and Derby Unitary Area at the time of the episode, because reliable mid-year population estimates were available for these areas. Mid-year population estimates were required to calculate rates.

### Prescriptions

We obtained data on prescriptions of the study drugs for individuals aged 15 years and over from the Clinical Practice Research Datalink (CPRD [20]), which is a governmental research service which provides anonymised primary care records that can be used for public health research. It currently includes data on just under 10% of the UK population. Patients included in the CPRD have been found to broadly represent the UK population in terms of age, sex and ethnicity [21]. Information from the CPRD included the number of patients registered with the general practices appearing in the CPRD database and the number of individuals who were prescribed the specific study medication, by individual year (2005–2012), age and gender.

### Statistical analysis

We calculated rates of non-fatal self-poisoning based on the number of episodes of non-fatal self-poisoning per 100,000 person-years using mid-year population estimates obtained from ONS for Oxford City, the City of Manchester, and Derby Unitary area for 2005–2012. We calculated rates of suicide by self-poisoning per 100,000 person-years using England’s mid-year population estimates for 2005–2012, which we obtained from the Office for National Statistics (ONS). Prescription rates were calculated as the number of individuals prescribed a specific medication per 100,000 person-years registered with the general practices in the CPRD during 2005–2012. All analyses were carried out using the Poisson distribution with exact 95% CIs.

Our measures of relative toxicity included *case fatality* and *fatal toxicity*. Case fatality was calculated by taking the number of deaths by self-poisoning in England during 2005–2012 attributed to a given study drug divided by the number of episodes of non-fatal self-poisoning involving this drug during the equivalent period from the Multicentre Study of Self-harm in England and standardising these ratios to the risk for lithium (for the mood stabilisers) and chlorpromazine (for the antipsychotics). Fatal toxicity was calculated using the number of deaths by self-poisoning in England during 2005–2012 attributed to a drug divided by the number of individuals prescribed this drug during the equivalent period and standardised to the risk for lithium (for the mood stabilisers) and chlorpromazine (for the antipsychotics). We were not able to calculate a fatal toxicity measure for clozapine because this drug is rarely prescribed via GP surgeries. For this drug we therefore relied on case fatality. Lithium and chlorpromazine were selected as reference drugs on the basis of long-term use (these are older drugs with a substantial history of use). Confidence intervals for relative toxicity were calculated using the Poisson distribution.

We conducted two sets of analyses for each of the toxicity indices (fatal toxicity and case fatality). First, we included only deaths involving single drugs (with or without alcohol). Second, we also included cases of deaths involving multiple drugs where the drug of interest was the first drug recorded by the coroner, on the basis that this drug was likely to have been the main cause of death.

We also calculated the proportion of individuals for whom alcohol was mentioned in the coroner’s report for each of the drugs investigated. Statistical analyses were carried out using Excel 2013 and Stata 14.1.

### Ethical approval

The monitoring systems for self-harm in Oxford and Derby have approval from local Health /Psychiatric Research Ethics Committees to collect data on self-harm for local and multicentre projects. Self-harm monitoring in Manchester is part of a clinical audit system, and has been ratified by the local Research Ethics Committee. All three monitoring systems are fully compliant with the Data Protection Act of 1998. All centres have approval under Section 251 of the National Health Services (NHS) Act 2006 (formerly Section 60, Health and Social Care Act 2001) to collect patient identifiable information without patient consent.

We also obtained specific Independent Scientific Advisory Committee (ISAC) approval from the CPRD (protocol number 14_064R2) and also from ONS to obtain mortality data.

## Results

### Mood Stabilisers

During the eight-year study period (2005–2012) there were 59 suicide deaths in England due to single-drug poisoning with mood stabilisers and a further 37 cases where more than one drug was listed as contributing to the death but the mood stabiliser under investigation was listed first (Table 1). Of these 96 deaths, 55% involved males and 45% females. These were from a total of 3083 poisoning suicide deaths occurring during the study period. Of the 59 deaths, 7% were in people age 15–24, 61% were in people age 25–54, and 32% in people age 55 or older. In 51 cases there was a verdict of intentional self-poisoning and in 45 cases a verdict of undetermined intent.

**Table 1:** Number and rates of suicides involving single-drugs or single and multiple (first-listed) drugs in England and of individuals prescribed each medication, in individuals aged 15 years and over (2005–2012)

	Single-drug deaths in England, total n	Single-drug deaths in England, death rate per 100,000 person-years (95% CI)	Single and first listed drug deaths in England, total n	Single and first listed drug deaths in England, Death rate per 100,000 person-years (95% CI**)**	Prescriptions, average number per year	Prescription rate per 100,000 person-years (95% CI)	Non-fatal self-poisonings in three centres, average number per year	Self-poisoning rate per 100,000, per 100,000 person-years (95% CI)
Mood Stabilisers								
Lithium	6	0.002 (0.001–0.004)	10	0.003 (0.001–0.005)	4956	107.24 (106.18–108.30)	12	1.64 (1.32–2.01)
Sodium valproate	15	0.004 (0.002–0.007)	18	0.005 (0.003–0.008)	15,433	333.97 (332.11–335.84)	32	4.45 (3.92–5.03)
Carbamazepine	33	0.010 (0.007–0.014)	56	0.016 (0.012–0.021)	18,836	407.62 (405.56–409.68)	28	3.87 (3.88–4.42)
Lamotrigine	5	0.001 (0.00–0.003)	12	0.004 (0.002–0.006)	7635	165.22 (163.91–166.54)	15	2.02 (1.67–2.43)
Total	59		96		46,860		87	
Antipsychotics								
Chlorpromazine	12	0.004 (0.002–0.006)	19	0.006 (0.003–0.009)	3760	81.37 (80.46–82.30)	25	3.44 (2.97–3.95)
Risperidone	1	0.000 (0.00–0.002)	2	0.001 (0.00–0.002)	8302	179.65 (178.29–181.03)	34	4.69 (4.15–5.29)
Quetiapine	40	0.012 (0.008–0.016)	65	0.019 (0.015–0.024)	9971	215.78 (214.28–217.28)	73	10.20 (9.39–11.07)
Olanzapine	55	0.016 (0.012–0.021)	78	0.023 (0.018–0.029)	10,568	228.70 (227.16–230.25)	61	8.55 (7.81–9.34)
Clozapine	35	0.010 (0.007–0.014)	40	0.012 (0.008–0.016)	157	3.39 (3.21–3.59)	4	0.54 (0.36–0.77)
Total	143		204		32,758		197	

During the same period, 687 non-fatal self-poisonings with mood stabilisers were identified through the Multicentre Study of Self-harm in England. Data taken from the CPRD (which contains records from approximately 10% of the GP surgeries in England) indicated that there were on average 46,860 prescriptions per year of the mood stabilisers under investigation during the 2005–2012 period (Table 1).

### Case fatality indices

Using single-drug deaths, carbamazepine appeared to be over twice as likely to result in death when taken in overdose compared to lithium, although the evidence was weak. There was no indication of major differences in toxicity for the other mood stabilizers (Table 2).

**Table 2:** Case fatality indices– number of suicides involving a given drug per non-fatal self-poisoning episode relative to a. lithium and b. chlorpromazine

	Odds ratio (95% CI)	
	Single drug only	Single and first listed drug
A. Mood Stabilisers		
Lithium	1.0	1.0
Sodium Valproate	0.92 (0.35–2.45)	0.66 (0.30–1.49)
Carbamazepine	2.33 (0.94–5.74)	2.37 (1.16–4.85)
Lamotrigine	0.68 (0.20–2.28)	0.97 (0.40–2.35)
B. Antipsychotics		
Chlorpromazine	1.0	1.0
Risperidone	0.06 (0.01–0.47)	0.08 (0.02–0.34)
Quetiapine	1.12 (0.58–2.18)	1.15 (0.67–1.97)
Olanzapine	1.84 (0.97–3.52)	1.65 (0.97–2.80)
Clozapine	18.53 (8.69–39.52)	13.38 (6.88–26.00)

The results were similar when we also included cases in which multiple drugs had been consumed in self-poisoning deaths but the drug of interest was listed first, except that the result for carbamazepine was statistically significant. This indicates that overdoses with carbamazepine were more likely to result in death than overdoses with lithium (Table 2).

### Fatal toxicity indices

Measures of fatal toxicity showed a similar pattern of results to those for case fatality, except that the finding for carbamazepine was attenuated and consistent with chance (Table 3). Using additional cases in which the drug of interest was listed first where multiple drugs had been ingested indicated the same pattern.

**Table 3:** Fatal toxicity indices – number of suicides involving a given drug per individuals prescribed this drug relative to a. lithium or b. chlorpromazine

	Odds ratio (95% CI)	
	Single drug only	Single plus first listed drug
A. Mood Stabilisers		
Lithium	1.0	1.0
Sodium Valproate	0.80 (0.31–2.07)	0.58 (0.27–1.25)
Carbamazepine	1.45 (0.61–3.45)	1.47 (0.75–2.89)
Lamotrigine	0.54 (0.17–1.77)	0.78 (0.34–1.80)
B. Antipsychotics		
Chlorpromazine	1.0	1.0
Risperidone	0.04 (0.01–0.29)	0.05 (0.01–0.21)
Quetiapine	1.26 (0.66–2.40)	1.29 (0.77–2.15)
Olanzapine	1.63 (0.87–3.05)	1.46 (0.89–2.41)

#### Antipsychotics

During the study period there were 143 suicide deaths due to single-drug poisoning with major tranquillisers and a further 61 in which there was evidence of multiple-drug ingestion but the drug of interest was listed first. Of these 204 deaths, 66% involved males and 34% females. These were from a total of 3083 suicides involving self-poisoning. In terms of age breakdown, 6% of deaths occurred in people aged 15–24 years, 80% in people aged 25–54 year and 14% of deaths in people 55 years or older. In 91 cases there was a verdict of intentional self-poisoning and in 113 cases an open verdict.

There were 1631 non-fatal self-poisoning episodes in the Multicentre Study of Self-harm in England in which antipsychotics were ingested. During the equivalent period, 32,758 individuals in the CPRD were prescribed the study antipsychotics yearly.

### Case fatality indices

The toxicity of risperidone was lower than the reference drug chlorpromazine. Clozapine appeared to be almost 19 times more toxic in overdose than chlorpromazine. Including deaths where the drug of interest was listed first in multiple drug deaths produced a similar pattern of results (Table 2).

### Fatal toxicity indices

The fatal toxicity approach using single-drug data also showed risperidone to have lower toxicity relative to chlorpromazine. Using cases where the drug of interest was listed first in multiple drug deaths produced the same pattern of results (Table 3). As noted earlier, the fatal toxicity approach could not be used for clozapine since complete data on prescribing were not available from the CPRD because most prescriptions are provided by hospitals.

### Alcohol

Alcohol was involved in very few of the deaths. Alcohol was mentioned in the coroner’s report in only five cases (8.5%) of single-drug deaths involving mood stabilisers, and in 22 deaths involving antipsychotics (15.4%). In neither drug group was there any indication of excess alcohol involvement with specific drugs.

## Discussion

We have examined the relative toxicity of individual mood stabilisers and antipsychotics using two measures of toxicity – case fatality and fatal toxicity. Both the case fatality and fatal toxicity approaches produced similar results. The more conservative method of including only deaths where one drug was listed on the coroner’s report was confirmed by a secondary analysis in which we also included cases with the involvement of more than one drug, but where the drug of interest was listed first. This second approach was used on the basis that the first drug was likely to have been the main cause of death. This supports the findings, as does the convergence of the results of both the fatal toxicity (not possible for clozapine) and case fatality methods.

### Mood Stabilisers

Perhaps the most notable finding regarding the mood stabilisers was that lithium, contrary to a tendency to regard this drug as particularly toxic in overdose [22] did not appear to be more toxic compared to the other mood stabilisers included in this study. This finding, together with increasing evidence that lithium may have specific anti-suicidal effects, should encourage clinicians to consider the use of lithium in patients with recurrent affective disorder, including those at risk of suicide.

There was some indication to suggest that carbamazepine may be more toxic relative to the other mood stabilisers, although evidence in support of this was shown in only one of the four models assessing the relative toxicity of carbamazepine. Carbamazepine, like most of the other mood stabilisers, is an anticonvulsant. While people with bipolar disorder have an elevated risk of taking prescribed drugs in overdose, [23] it is also important to recognise that risks of both suicide and non-fatal self-poisoning are elevated in people with epilepsy [8, 24]. Although antiepileptic drugs in general may not lead to higher rates of suicidal behaviour, carbamazepine has been found to be associated with increased levels of suicide attempts compared to other anti-epileptics [25] and carbamazepine is related to a higher risk of suicide attempts in bipolar disorder [26]. Carbamazepine also interacts with other drugs, which may increase its toxicity [27].

Neither of the other drugs (sodium valproate or lamotrigine) showed any sign of elevated toxicity relative to the reference drug.

### Antipsychotics

In this analysis, clozapine was the most toxic of the drugs examined, although only the case fatality approach could be used to assess its relative toxicity because clozapine is prescribed mainly from hospitals. Clozapine is an atypical antipsychotic usually used to treat patients with schizophrenia who have not responded to other antipsychotics [28]. Its use is already tightly regulated in the UK, [29] mainly because of the risk of blood dyscrasia [30]. Clozapine is often used to treat serious psychotic disorders of patients who have not responded to treatment with other drugs; patients who may already be at higher risk of suicide. However, in one major trial clozapine was found to reduce suicidal behaviour in people with schizophrenia who were at high risk of suicide, [31] a result which has support from longitudinal analysis of patient records on and off clozapine. [32] This provides support for the suggestion that suicide prevention could be an additional indication for clozapine. It is approved for this indication in the USA. [33] Nevertheless, the high toxicity of the drug in overdose needs to be considered in management. There should be careful monitoring for possible suicidal ideation and possibly controlled availability of the drug and regular monitoring of mood and suicidal ideation where risk is thought to be elevated.

Risperidone was also found to have lower toxicity than the reference drug chlorpromazine using both case fatality and fatal toxicity approaches, while both olanzapine and quetiapine were not found to be more toxic than the reference drug. These findings should be taken into account when prescribing antipsychotics.

### Strengths and limitations

In this study we used two methods to evaluate the relative toxicity of mood stabilizers and antipsychotics (case fatality and fatal toxicity), the results of which largely converged. Findings from the primary analysis using only deaths attributed to a single drug were consistent with findings using multiple-drug deaths, where the drug of interest was listed first in the coroner’s report. These analyses were based on several large datasets, including all the deaths in England attributed to self-poisoning between 2005 and 2012. Another strength is the consistent findings between the results of the fatal toxicity and case fatality approaches, although only the latter approach was possible for clozapine.

There are also several limitations to this work. Perhaps most importantly, our data were taken from different sources. The mortality data were obtained from ONS and covered the entire population of England, prescription data were provided by the CPRD and comprised medical records for just under 10% of the UK population, and data on non-fatal self-poisoning were obtained from the Multicentre Study of Self-harm in England, which covers three cities in England. However, these last two sources provided relatively large datasets and are likely to be reasonably representative of the population as a whole [21].

We also have limited data on the cause of death. There is no information on dosage of drugs taken in overdose or relative blood levels of drugs (in the cases where multiple drugs were taken). Therefore we cannot draw firm conclusions on drug interactions. However, this was mitigated by using single-drug deaths in our primary analyses. Similarly, we only have information on the reported presence or absence of alcohol in the bloodstream but no information on alcohol levels and hence the contribution of alcohol to deaths. We included deaths of undetermined intent (open verdicts) in line with current UK policy on suicide research. This is because the majority of deaths with this verdict are probable suicides, [16, 15] although it is not possible to be certain about intent in such cases.

Data on non-fatal self-poisoning was taken from data collected from emergency departments. It is important to note that self-harm in the community frequently does not result in presentation to an emergency department, although information from studies of young people indicate that hospital presentation is more likely where the method of self-harm is self-poisoning [34, 35]. It should nonetheless be acknowledged that the number of non-fatal poisonings will be an underestimate. Of course, this problem does not affect the relative toxicity findings.

It is possible that differences in patient groups may have contributed to the observed findings. For example, patients treated with lithium are likely to have bipolar disorder, while those treated with anticonvulsants may have a range of disorders including bipolar, epileptic or borderline personality disorders. The patients in each group may therefore differ in their risk of suicide or self-harm and access to potentially lethal doses of medications. As well, certain drugs prescribed for a given disorder may be a more effective treatment but also more toxic in overdose, meaning that it may be difficult to interpret the risks and benefits using this approach.

## Conclusions

The finding that lithium did not appear to have elevated toxicity when used for self-poisoning and that very few deaths occurred due to lithium ingestion in overdose may suggest the potential value of lithium as an anti-suicidal agent in people with mood disorders. The possible excess toxicity of carbamazepine in overdose compared to the other mood stabilisers should be borne in mind when prescribing, especially in patients with bipolar disorder or epilepsy thought to be at risk of suicidal behaviour.

Among the antipsychotics, clozapine is clearly far more toxic than other drugs. This adds to the need for very careful management when treating patients who are prescribed this drug, especially if they are considered to be at risk of suicidal behaviour. Risperidone was found to have lower toxicity than the reference drug chlorpromazine.

## Abbreviations

CPRD: Clinical Practice Research Datalink
GP: General practice
ICD-10: 10th revision of the International Statistical Classification of Diseases and Related Health Problems
ISAC: Independent Scientific Advisory Committee
NHS: National Health Service
ONS: Office for National Statistics
OR: Odds ratio
UK: United Kingdom
USA: United States of America
